# Mixed Matrix Membranes Adsorbers (MMMAs) for the Removal of Uremic Toxins from Dialysate

**DOI:** 10.3390/membranes12020203

**Published:** 2022-02-09

**Authors:** Matilde De Pascale, Maria Grazia De Angelis, Cristiana Boi

**Affiliations:** 1 Department of Civil, Chemical Environmental and Materials Engineering, DICAM, University of Bologna, Via Terracini 28, 40131 Bologna, Italy; matilde.depascale@gvs.it; 2 Institute for Materials and Processes, School of Engineering, University of Edinburgh, Sanderson Building, Robert Stevenson Road, Edinburgh EH9 3FB, UK; 3 Italian Consortium for Science and Technology of Materials (INSTM), 50121 Firenze, Italy

**Keywords:** porous mixed matrix membrane adsorbers, uremic toxins, urea, zeolite, activated carbon, water purification, dialysis

## Abstract

We developed Mixed Matrix Membrane Adsorbers (MMMAs) formed by cellulose acetate and various sorbent particles (activated carbon, zeolites ZSM-5 and clinoptilolite) for the removal of urea, creatinine and uric acid from aqueous solutions, to be used in the regeneration of spent dialysate water from Hemodialysis (HD). This process would allow reducing the disproportionate amount of water consumed and permits the development of closed-loop HD devices, such as wearable artificial kidneys. The strategy of MMMAs is to combine the high permeability of porous membranes with the toxin-capturing ability of embedded particles. The water permeability of the MMMAs ranges between 600 and 1500 L/(h m^2^ bar). The adsorption of urea, the limiting toxin, can be improved of about nine times with respect to the pure cellulose acetate membrane. Flow experiments demonstrate the feasibility of the process in a real HD therapy session.

## 1. Introduction

### 1.1. Motivation

Chronic kidney disease (CKD) is a debilitating condition in which the kidneys are no longer able to remove uremic toxins, salts and excess fluids from human blood and life support treatments such as Hemodialysis (HD) or Peritoneal Dialysis (PD) are required. In 2017, the global prevalence of CKD was 9.1% with 697.5 million cases that resulted in 1.2 million deaths and 35.8 million people with disability-adjusted life years. CDK increased by 29.3% since 1990, mostly due to ageing of the world’s population [1].

The CKD burden is particularly high in Oceania, sub-Saharan Africa and Latin America, highlighting the need to develop more affordable therapies that can be accessed worldwide [2]. Furthermore, the current HD process has a huge environmental impact, as almost 500 L of tap water are used to obtain about 120 L of ultrapure water via Reverse Osmosis (RO) for each HD session [3]. Reducing the water consumed in the HD process would be an important step for reaching the UN sustainable development goals. Several studies proposed the reuse of dialysate water in landscape and irrigation programs [4,5,6] or the utilization of reject RO water for sanitation [7,8]. However, the optimal solution would be to purify the spent dialysate and recirculate it through the dialysate side of the HD, making the process circular and allowing a reduction the volume of water involved as to enable home dialysis devices such as Wearable Artificial Kidneys (WAK). The development of WAK would reduce the issues associated to the discontinuous hospital treatment of patients, such as hypertension, heart disease, mortality, low life quality. For the development of a small device like WAK, it is essential to regenerate efficiently the dialysate from patients in a closed-loop system, allowing to reduce the volume of dialysate to below 0.5 L [9].

The REcirculation DialYsis (REDY), popular in the 1970s–1990s, was the only commercial system aimed at recycling dialysate water [10]. It was a transportable sorbent system made by several layers: Activated Carbon (AC) which adsorbs non-urea organic compounds, urease immobilized onto alumina to convert urea into ammonium and (bi)carbonate, zirconium phosphate ion-exchanger to exchange ammonia for sodium or hydrogen and zirconium oxide and zirconium carbonate to adsorb phosphate in exchange for hydroxide, bicarbonate and acetate [10]. The system was abandoned due to several issues associated with the use of urease and activated carbon, which could release aluminum ions, toxic for patients [9]. Second generation systems inspired by REDY are still under development and some of them reached the clinical trials stage. However, none has been commercialized yet, mostly due to issues related to the presence of urease [11,12,13].

In this work we tackle the purification of dialysate with a different concept, the Mixed Matrix Membrane Adsorber (MMMA), which was originally proposed for protein separation [14]. In such systems, particles with the ability to capture uremic toxins embedded into a porous polymeric matrix may increase the toxin removal and reduce the leakage of toxic compounds to the recycled dialysis water. The membrane pores minimize friction losses, maximize the flux and leave the sorbent particles available for toxins removal. These membranes can be inserted into a low-pressure purification column like the ones used in membrane chromatography, and they are easy to scale-up and processed in different shapes such as flat sheets, hollow fibers, monoliths etc. to optimize the fluid dynamics. Furthermore, one can stack different membrane layers with specific removal properties in a column by varying the polymer nature and structure and the type of sorbent. Finally, the materials are only in contact with the dialysate water and not with blood, thus widening the number of possible materials to be adopted.

In the following section we provide an overview of the main issues and solutions associated with the removal of uremic toxins in dialysis and correlated processes, and we explain how our system fits in this landscape.

### 1.2. Uremic Toxins Removal

The solutes that need to be removed to regenerate dialysate include ions, such as phosphate and potassium, which can be removed by ion-exchangers, and uremic toxins. Uremic toxins are normally divided into three groups: (i) low molecular weight solutes that are free in solution such as urea, uric acid or creatinine; (ii) high molecular weight protein-bound solutes such as p-cresol and indoxyl sulphate and (iii) middle molecular weight peptides [15].

Several studies indicate that the biggest challenge for dialysate regeneration is the removal of small uremic toxins, and in particular urea [16]. It must be also observed that, although it is out of the scope of the present paper, that current HD membranes do not remove the totality of the uremic toxins, especially the larger ones that do not diffuse through membrane pores [17].

#### 1.2.1. Sorbents

Adsorption was considered from the very early stages of WAK development as the elective process for regenerating the spent dialysate, with activated carbon considered one of the best materials for its ability to remove most solutes [18,19]. However, the removal of urea is relatively low and other systems such as enzymatic hydrolysis, electrochemical oxidation (EO), chemisorption and physisorption were explored [20].

Wernert et al. tested zeolites such as Linde Type A (LTA), stilbite (STI), silicalite (MFI), mordenite (MOR) and faujasite (FAU) [21]. Using solutions at initial concentrations close to that of patients with renal failure, it was possible to remove 75% of creatinine by adsorption onto acidic MOR and 60% of p-cresol by adsorption onto a hydrophobic MFI, while urea and uric acid are efficiently adsorbed by STI [21].

Bergé-Lefranc et al. studied the adsorption of p-cresol and creatinine from aqueous and physiological solution onto MFI and MOR45 zeolites [22,23]. They compared the performance of ZSM-5 zeolites and of a pure silicalite. ZSM-5 zeolites differ for the charge compensating cations, and for the different synthesis pathways. MFI-type zeolites adsorb 85% of p-cresol in the uremic concentration range. Cheah et al. observed that mesoporous silica and amine-functionalized mesoporous silica have a faster urea adsorption rate and a superior binding capacity with respect to activated carbons [24].

Harm et al. showed that the pore size of the sorbent determines its selectivity towards the toxins, so that the performance of an adsorbent can be adjusted for the treatment by tailoring its pore size distribution [25].

Pavlenko et al. hypothesized that carbon-based sorbents with two well-defined pore sizes, CMK-3, could remove the daily production of toxins in kidney failure patients [26].

Cheng et al. explored the adsorption of p-cresol, creatinine and urea onto AC, three types of zeolite ZSM-5, and graphene oxide (GO) [27]. The ZSM-5 with the highest Si/Al ratio (400), and it is also the less hydrophilic, is the most effective for urea removal (0.7 mmol/g), while GO has the highest adsorption capacity for p-cresol and creatinine (4.01 and 1.01 mmol/g, respectively).

Metal-Organic Frameworks (MOFs) can also be exploited as adsorbents for artificial kidney applications. Yang et al. tested MIL-100(Fe), a MOF which has a maximum adsorption of creatinine of 190.5 mg/g in a phosphate-buffered saline solution [28].

Wernert et al., who studied the adsorption of p-cresol onto commercial hemodialysis membranes based on polysulfone, cellulose diacetate and polyacrylonitrile, proved that uremic toxins may also be adsorbed onto the polymeric material of the membrane and not only diffuse through their pores [29].

In the last years, other sorbents such as molecularly imprinted polymers were developed, but for a more complete overview of the materials used to remove uremic toxins from blood, and of their potentialities and disadvantages, we address the reader to the recent review of Ma and coauthors [19]. 

#### 1.2.2. Polymeric Membranes

Although porous polymeric membranes are used routinely in hemodialysis for blood purification, providing pores that allow uremic toxins to be transferred from blood to the dialysate side, they were rather seldom considered to purify and recycle the spent dialysate water. In the early work by Kraus et al. the dialysate water from peritoneal dialysis was purified through different polymeric membranes used in reverse osmosis such as cellulose acetate and polyamides, with the purpose of developing mini artificial kidneys [30]. The membranes reached urea rejections as high as 96 % and even higher removal of creatinine and uric acid at 50 atm. A more sophisticated system based on an integrated membrane/sorbent layout was also applied to a PD-based WAK system, where sorbents were placed in the shell-side of a hollow fiber dialyzer [31].

Abidin and coauthors fabricated dual layer hollow fiber (DLHF) membranes consisting of a Polysulfone (PS) inner layer and a PS/amino-silanized poly(methyl methacrylate (N-PMMA) outer layer that exhibited a static adsorption capacity of up to 27.6 mg/g and 39.2% urea removal in dynamic filtration conditions [32]. The proposed process is that of combined membrane adsorption and filtration for the regeneration of dialysate.

#### 1.2.3. Mixed Matrix Membranes (MMMs)

In recent years the concept of combining polymers and sorbents to fabricate Mixed Matrix Membranes was applied to the removal of uremic toxins in view of blood purification. Mixed matrix membranes are composite materials in which sorbent particles are dispersed into a polymeric matrix and can efficiently combine the selective and transport properties of the two phases.

In the following, to avoid confusion, we will refer to Mixed Matrix Membranes (MMMs) as composite materials used in filtration or dialysis in a continuous mode of operation. This is to distinguish them from Mixed Matrix Membrane Adsorbers (MMMAs) which are discussed in the following section. MMMAs have a structure similar to MMMs, but they are applied in a discontinuous purification process similar to adsorption chromatography.

The working principle of the two types of membranes is similar though: the polymer membrane guarantees high flux and low pressure drop, provides a high contact area and can also contribute to separation due to sorption and diffusion of the various toxins in it [29]. The main contribution to toxin removal is given by the adsorbent particles, which permanently capture the toxins, with capacities depending on the size and chemical nature of the toxin. The process is intrinsically discontinuous as the particles eventually reach saturation.

The use of MMMs in HD has been proposed by several authors in different works, which mostly targeted blood purification rather than dialysate regeneration. However, the two processes have many aspects in common and it is worth recalling the main results obtained, especially because most studies were actually carried out using uremic toxins dispersed in water as model system, which is the same situation encountered in dialysate regeneration.

The aim of adding sorbent particles to a polymeric material in blood purification is to enhance the removal capacity towards all kinds of uremic toxins, including the larger ones. The process exploits the solute convection through the porous membranes and their diffusion into the porous particles, followed by adsorption onto the sorbent surface. Since the materials must be compatible with blood, which is not the case, for instance, of activated carbon, this approach limits the range of materials that can be used.

Tijink et al. dispersed AC particles on the dialysate side of a dual layer polyether sulfone dialysis membrane, concluding that the accessibility and removal capacity of the AC particles to creatinine and the protein-bound solute para-aminohippuric acid (PAH) by MMMs is the same as that measured during AC adsorption [33]. Dual layer porous membranes of cellulose acetate were built with activated carbon to circumvent the problem of AC blood compatibility. The layer in contact with blood was made of pure CA while the dialysate side was based on AC particles embedded onto the CA matrix [34]. The membranes were tested in a simplified dialysis experiment for the removal of creatinine and showed a high removal capacity for this toxin, mostly due to the adsorption contribution. The authors proposed these membranes in different blood purification treatments, i.e., hemoperfusion, hemofiltration and hemodialysis.

This concept, proven in flat sheet membranes, was later extended to hollow fibers (HF) HD membranes based on polymers and AC [35], that removed 57% of p-cresyl sulfate, 82% indoxyl sulfate and 94% of hippuric acid from spiked human plasma in 4 h in static conditions. Geremia et al. demonstrated that AC/poly ether sulfone/polyvinyl pyrrolidone MMM can achieve endotoxin-free dialysate together with high removal of uremic toxins from human plasma, thus reducing the risk of transferring inflammatory agents to the patient blood [36]. Ter Beek et al. showed that, when MMM hollow fibers are used in outside-in filtration mode their removal capacity towards protein-bound uremic toxins is higher [37].

Fahmi et al. incorporated graphene oxide into a flat sheet asymmetric PES membrane, enhancing the membrane capacity to remove creatinine from water [38]. Fu et al. fabricated porous polyether sulfone MMMs incorporating AC, ZSM-5, and GO via non-solvent-induced phase inversion and tested them for p-cresol and creatinine removal from simulated serum [39]. In a closed-loop dynamic experiment at 37 °C, the AC-incorporated membranes removed 80.0% of p-cresol and 7.4% of creatinine within 4 h.

Electrospinning was also applied to produce nanofibers with the ability of removing uremic toxins: Lu et al. prepared electrospun nanofibrous polyacrylonitrile membranes with zeolite ZSM-5 particles for creatinine removal [40,41], while Namekawa et al. developed a zeolite-poly(ethylene-co-vinyl alcohol) composite nanofiber membrane to remove creatinine for blood purification [42]. Haghdoost et al. fabricated core–shell structured composite nanofibers by electrospinning two polymers and two types of zeolite in a coaxial system, obtaining selective capability for creatinine [43]. However, nanofibrous membranes for this application are at an earlier stage of development compared to traditional membranes, and their low mechanical resistance can still be an issue, although their application in biomedical related applications is extremely promising.

#### 1.2.4. Mixed Matrix Membrane Adsorbers

The idea was introduced in 2003 by Avramescu et al., who developed a mixed matrix membrane adsorber for the separation of two proteins of similar size, albumin and hemoglobin [14]. The membranes were tested in a membrane chromatography configuration, that is alternative to fixed bed chromatography for the purification of biomolecules and can offer larger surface areas, short diffusion paths and low pressure drops. The composite matrices, made of ion-exchange resin beads dispersed into a porous polymeric matrix of ethylene–vinyl alcohol copolymer (EVAL), showed high static and dynamic protein adsorption capacities. Dynamic experiments were carried out with a layered stack of 10 membranes at low pressure [14]. The developed materials combined the advantages of membrane technology (easy scale-up, low-pressure drop) with the high binding capacity of ion-exchange resins, and are resistant to fouling. Subsequent works regarded the removal of bilirubin from plasma, where the adsorptive membrane was modified with bioligands. The concept was also applied to blood purification by Saiful, who fabricated a MMMAs with 60 wt% of AC in cellulose acetate for creatinine removal in batch adsorption and dynamic adsorption in 3 different operational modes, namely dead-end filtration, cross flow filtration and dialysis [44]. In the first case the process is similar to the one proposed in the present study, although we are aiming at dialysate water regeneration rather than blood purification as in the case of Saiful. Furthermore, the dynamic adsorption data were presented only for one loading of activated carbon (60 wt%) and the effect of varying filler concentration on the performance was not assessed.

### 1.3. MMMAs Developed in This Work

The choice of the polymeric material used in this study, cellulose acetate, was motivated by its relatively low cost, easy processability and higher water flux compared to other urea-rejecting polymers used in desalination like polyamides [30]. Blood compatibility is not an issue in our case as we are targeting dialysate water treatment.

The choice of sorbents was based on the idea of testing materials with different microstructure and chemical nature, selecting those with a proven ability to remove uremic toxins. Activated carbon was a natural choice due to its high degree of porosity and high surface area. The other sorbents considered were two different types of zeolites which can offer selectivity towards urea due to their chemical nature and specific interactions. In general, zeolites have a uniform pore size and tunable physical and chemical properties. However, they are easily dissolved in dialysate as they are hydrophilic; for this reason, their incorporation in a polymer matrix is essential for their stabilization.

ZSM-5 is a well-known synthetic zeolite with a tunable Si/Al ratio that allows to modify its hydrophilicity. Previous studies that applied this sorbent to the removal of uremic toxins showed that the highest Si/Al ratio commercially available (400) is the best in terms of urea removal capacity [27]. A further dealumination treatment was applied in this work to make the material even more hydrophobic, as detailed in the following.

The other zeolite chosen is a clinoptilolite that belongs to the heulandite (HEU) framework, labelled here with its commercial name ZUF (Zeolite Ultra Fine). It is a natural zeolite commercialized as a medical product with detoxifying properties thanks to its ability to remove metals and toxins from the organism in the gastrointestinal duct [45]. Previous studies indicate that this material has a selective ability for urea under ambient conditions from either aqueous or ethanolic solutions [46]. Proposed applications of this material are indeed in the development of urea sensors [47] or controlled-release urea fertilizers [48]. However, to our knowledge this material has never been evaluated before for use in uremic toxins removal in relation to treatment of renal disease.

For both the zeolites considered, the pore size varies from 0.5 to 1 nm, making them very promising for the removal of low molecular weight solutes.

## 2. Materials and Methods

### 2.1. Materials

#### 2.1.1. Chemicals and Fillers

Urea was purchased from Sigma Aldrich (Saint Louis, MO, USA) while creatinine and uric acid from Acros Organics (Geel, Belgium). The base polymer for membrane preparation was cellulose acetate (CA), purchased from Sigma Aldrich with an average molecular weight, Mn of 30,000 Da, a degree of acetyl substitution approximately of 2.4 and a density of 1.3 g/mL at 25 °C.

Poly(ethylene glycol) (PEG) of 400 Da by Sigma Aldrich (St. Louis, MO, USA) was used as pore former, while 1-methyl-2-pyrrolidone (NMP) purchased by Fluorochem (Hadfield, UK) was used as solvent. NMP has a very high boiling point, around 202 °C and a density of 1.03 g/mL.

Water, HPLC grade was purchased from Merck (Darmstadt, Germany) and Acetonitrile supplied by Scharlau (Barcelona, Spain).

#### 2.1.2. Uremic Toxins

Urea, creatinine and uric acid, the uremic toxins, present at the highest concentration in the blood of patients with end stage renal disease, were considered in this research. They are classified as soluble compounds with low molecular weight, MW < 500 Da, and as non-protein-bound toxins. For each of them, we used, as operative limits, the value of concentration present in the blood of healthy individuals, c_N_, and the highest concentration possible in hyperuricemia conditions, c_MAX_. The characteristics of the toxins are listed in Table 1. The toxin concentration was measured with UV readings at 200 nm for urea, at 235 nm for creatinine and at 290 nm for uric acid using a Shimadzu UV-1601 UV-Visible spectrophotometer.

#### 2.1.3. Adsorbents

The activated carbon used (Darco^®^KB-WJ, Sigma Aldrich) has a high surface area of 1857 m^2^/g, a total pore volume of 1.49 cm^3^/g and a mesopore volume fraction of 0.59 [49]. Only the fraction of material with a particle diameter < 53 µm was used for the experiments.

**Figure 1:**
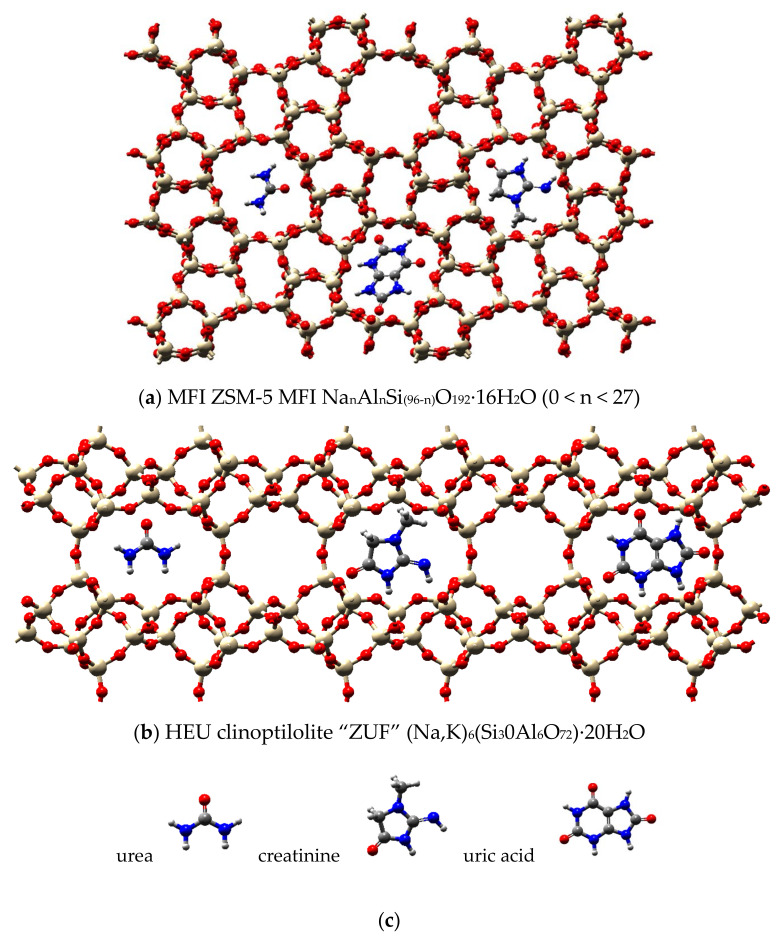
Molecular structures of the zeolite crystals (**a**) ZSM-5; (**b**) ZUF (clinoptilolite) Reported for reference also the structures of the uremic toxins considered (**c**).

Two types of zeolites, ZSM-5 and ZUF, were tested, and their structure is reported in Figure 1. Pellets of ZSM-5 with an initial Si/Al ratio of 400 [50] (ZEOcat^®^ Z-400 kindly provided by ZeoChem, Uetikon, Switzerland) were initially ground. The surface area of this adsorbent is estimated to be 280 m^2^/g, the total pore volume is 0.24 cm^3^/g with 92% of mesopores [51]. Only the fraction of material with a particle diameter < 150 µm was taken into consideration for the experiments.

The HEU clinoptilolite ZUF in powder (Zeolith-Bentonit-Versand.de, Chemnitz, Germany) has a particle diameter lower than 10 µm as written in the material datasheet. The Si/Al ratio for clinoptilolites was reported to be higher than 4 and the BET surface area 84.45 m^2^/g, with a total pore volume of 0.053 cm^3^/g and a fraction of micropores of 2.54% [52].

Each adsorbent in powder form was analysed to determine the particle size distribution. For the activated powdered carbon, minced with a grinder, a wet sieving procedure was required to avoid electrostatic interactions between the particles and the metal wire of the sieve. The sieves had different mesh size: 106, 90, 75, 63 and 53 μm. The dried samples were weighted, and the results were plotted to identify the amount of carbon obtained for each fraction. For the zeolites, a Laser Particle Sizer Analysette^®^ 22 (Fritsch) suitable for solid suspensions with particle size from 0.16 µm to 1100 µm was used.

#### 2.1.4. Zeolites Activation

Previous works have underlined that the removal capacity of zeolites towards uremic toxins is improved by an acid dealumination treatment [53]. According to the literature, acid activation with 1 M HCl should enhance the adsorption capacity of the materials by unblocking the channels of alumino-silicate framework structure of the zeolite [52,54]. The zeolites were washed with demineralized water and dried in the oven at 110–120 °C for 2–3 h. Subsequently, 5 mL of 1M HCl were added for each gram of material. The suspension was stirred for 48 h at 90 rpm, then filtered and washed several times to bring the pH back to neutrality. Finally, the wet powder was dried at 110 °C.

### 2.2. Membranes

#### 2.2.1. Pure Polymeric Membranes Preparation

Cellulose acetate membranes were prepared through the phase inversion casting technique [55]. A solution of NMP containing 13.5 wt% of CA and 8wt% of PEG 400 was prepared and stirred overnight to guarantee a good mixing between the different components. The membranes were casted with a knife with adjustable thickness, BYK-Gardner 2326. After casting, the membranes were immediately immersed in a deionized water bath at 23 °C, that was utilized as non-solvent for the phase inversion. The synthesized membranes were heat-treated for one week in a deionized water bath at 40 °C to remove the excess NMP and PEG.

#### 2.2.2. MMMAs Preparation

MMMAs were prepared according to a procedure similar to the one used for the CA membranes described above. Different amounts of ZUF (5 to 60 wt%), ZSM-5 (5 to 30 wt%) and AC (5 to 30 wt%) were soaked into NMP and sonicated until a homogenous dispersion was obtained [38]. Subsequently, 10% of the total amount of polymer was added to the solution and stirred up to complete dissolution to prevent aggregation of the zeolite particles. The rest of the polymer was finally adjoined, and the solution was stirred overnight. The casting procedure and the post-casting heat treatment were the same as in Section 2.2.1. The MMMAs prepared had a thickness of approximately 250 µm. The membranes were labelled according to the amount (in wt%) and type of filler added. For instance, MMMA_ZUF-5 is the membrane with 5 wt% of ZUF.

### 2.3. Membrane Characterization

#### 2.3.1. Morphological Analysis

The membrane morphology was studied by Scanning Electron Microscopy (SEM). Images were taken after metallization with aluminum by a Philips XL 20 instrument (Philips, Eindhoven, Netherlands). The presence of zeolites in the MMMAs was demonstrated, when they were not clearly detectable from the SEM images only, through Energy Dispersive X-ray Spectroscopy (EDS) spectroscopy using an EDS Philips Edax (Philips, Eindhoven, Netherlands).

#### 2.3.2. Water Uptake and Density

The membrane water uptake was determined at ambient condition by gravimetric measurements with an analytical balance (Mettler Toledo, precision 10^−4^ g). The water uptake, *m_w_*, was calculated as the difference between the hydrated membrane weight, *m_m,h_* measured after soaking the membranes for 2 d in deionized water, and the dry weight *m_m_* measured quickly after drying the membranes in vacuum at 100 °C overnight.

The density of the MMMAs, *ρ_m_,* was measured, by using a Density Kit MS-DNY 54 (Mettler Toledo) installed on the analytical balance. The system uses the Archimedes principle to estimate the volume displaced by the membrane by measuring the apparent weight of the sample in air (*A*), and in an auxiliary liquid with known density (*B*). As the dry MMMA is too unstable for a reliable measurement of this kind, we measured the density of the hydrated membrane instead (*ρ_m,h_*), using ethylene glycol as auxiliary liquid. The density of the hydrated membrane is then calculated as follows:

(1) $$\rho_{m,h}=\frac{A}{A-B}\left(\rho_{w}-\rho_{a}\right)+\rho_{a}$$

where *ρ_w_* and *ρ_a_* are the density of water and air in the experimental conditions. From the value of *ρ_m,h_* one can easily estimate the dry membrane density *ρ_m_* considering the additivity between the volumes of water and polymer in the hydrated membrane:

(2) $$\frac{m_{m,h}}{\rho_{m,h}}=\frac{m_{w}}{\rho_{w}}+\frac{m_{m}}{\rho_{m}}$$

Which can be rewritten as:

(3) $$\rho_{m}=\frac{\rho_{m,\;h}\rho_{w}m_{m}}{m_{m,\;h}\rho_{w}-\rho_{m}\left(m_{m,h}-m_{m}\right)}$$

#### 2.3.3. Dynamic Contact Angle

MMMAs dynamic contact angles were measured with a force tensiometer Sigma 700 (Biolin Scientific, Västra Frölunda, Sweden). MMMAs samples were cut in rectangular pieces and hung to an ultraprecise micro-balance while a lift with an extreme high resolution moves up and down a container with the liquid of interest (water). During the dipping phase, the advancing contact angle is measured, while the receding contact angle is measured during the de-wetting process.

#### 2.3.4. Hydraulic Permeability

The membrane permeability was obtained by measuring the pure water flux though a membrane disc of 6.35 cm diameter using an Amicon^®^ stirred cell (EDM Millipore, Bedford, MA, USA). A schematic drawing of the equipment used is reported in Figure S1a. Tests were performed at different pressures ranging from 0.5 to 1.5 bar. The hydraulic permeability was calculated with Darcy’s law:

(4) $$Q=L_{p}\;A\;\Delta P$$

where Q is the flow rate in L/h, A is the cross-sectional area in m^2^, ΔP is the transmembrane pressure in bar and L_p_ is the hydraulic permeability in L/(h m^2^ bar).

### 2.4. Adsorption Properties Characterization

#### 2.4.1. Batch Characterization of the Adsorbents

The binding kinetics of the different toxins was measured for all the three adsorbents considered. A sample of 1.25 g of adsorbent was immersed in a beaker containing 10 mL of the pure toxin solution and kept under mechanical agitation by the action of an orbital shaker. The toxin concentration was measured at regular time intervals by sampling the supernatant obtained after centrifugation of the suspension. The batch characterization of the adsorbents was completed by measuring the static binding capacity at equilibrium: 0.125 g of adsorbent were immersed in 4 mL of pure toxin solution at different concentrations and kept under agitation at 90 rpm for 24 h. All experiments were performed at room temperature.

#### 2.4.2. Batch Characterization of the Membranes

The static binding capacity of the membranes (pure CA and all MMMAs) for the uremic toxins was determined in experiments analogous to the ones performed with the pure filler. Two membrane disks of 15 mm of diameter were immersed in 2 mL of the pure toxin solution at *c_MAX_* and kept under agitation for 24 h before analysis.

#### 2.4.3. Continuous Removal Capacity for Single-Toxin Solutions and for a Toxin Mixture

Continuous adsorption tests were performed to calculate the dynamic binding capacity of the MMMAs. Membrane discs of 25 mm diameter were packed in a stainless-steel column that was connected to an FPLC system, Aktä Purifier (Cytiva, Milan, Italy).

Tests with the toxin mixture were performed using a layered stack of 22 membranes with a total bed height of 5.5 mm, corresponding to 2.7 mL of column volume (CV). The adsorption experiments were conducted at 3 different feed flow rates, namely at 0.5, 5 and 10 mL/min. Since the UV-Vis signal of creatinine and uric acid interferes with the one of urea, the toxin concentration in the flow-through was measured by HPLC analysis of the collected fractions. A XBridge C18 column purchased from Waters (Milan, Italy) was run in isocratic mode with a water/acetonitrile (98:2) mobile phase, a percentage that allowed to have the best peak resolution. A schematic of the apparatus is reported in Figure S1b.

## 3. Results and Discussion

### 3.1. Materials and Membranes Characterization

#### 3.1.1. Adsorbents Particle Size Analysis

The complete results of the granulometric analysis are reported in the Supplementary Materials, Figure S2. The data show that for activated carbon the fraction remaining after grinding and sieving is the one with diameter ≤ 53 µm. For both zeolites, a mean particle size of around 10 µm was obtained, while for the zeolites primed with HCl a reduction in the number of particles with size smaller than 10 µm after the treatment was observed.

#### 3.1.2. Membrane Morphology

**Figure 2:**
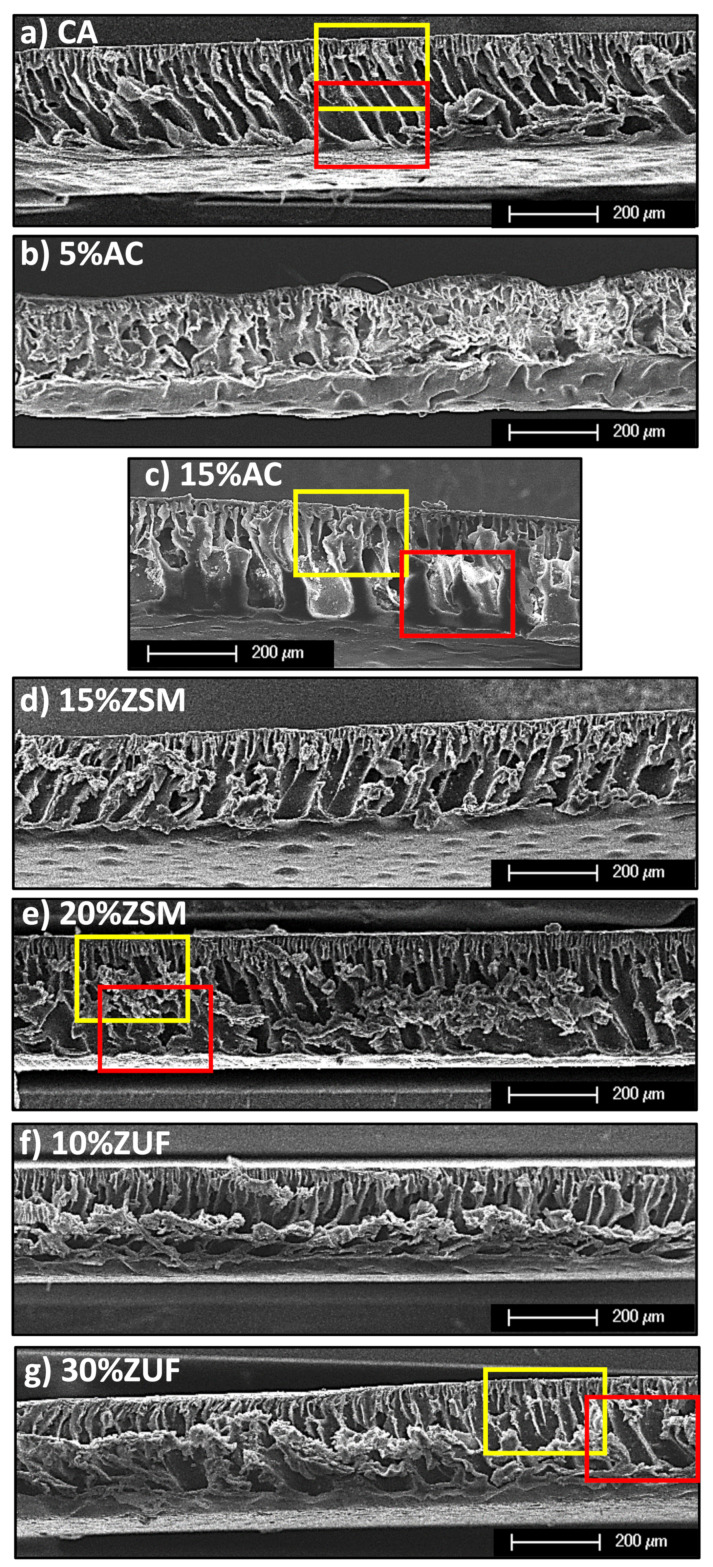
SEM images of the membranes cross section. (**a**) pure CA membrane; MMMAs containing (**b**) 5% of AC; (**c**) 15%AC; (**d**) 15% ZSM-5; (**e**) 20% ZSM-5; (**f**) 10% ZUF; (**g**) 30% ZUF.

In Figure 2 we can see a picture of the cross section and surface of the different membranes at a low magnification level. Images of this type are useful to assess the effect of the fillers on the overall morphology of the membrane, on its surface and pore distribution. The neat cellulose acetate membrane (Figure 2a) has a regular structure with finger-like parallel pores and a flat surface. The pores are well defined and evenly distributed along the membrane, and it is possible to distinguish the surface that was in contact with the air during casting (thin layer with small pores), and the surface that was in contact with the glass plate (thick layer with big channels).

The addition of activated carbon (5% and 15%, Figure 2b,c) disrupts the regular porous structure of the CA membrane and makes the surface corrugated. On the other hand, the addition of the crystalline fillers, ZSM-5 and ZUF, (Figure 2d–g) does not alter significantly the overall structure of the pores, but the filler tends to aggregate at the bottom of the membrane due to higher density of the MMMAs dope compared to the plain cellulose acetate and to activated carbon. Zeolite particles had a smaller average size compared to activated carbon, which can also contribute to justify the lower impact of this filler on the porosity and pores shape.

**Figure 3:**
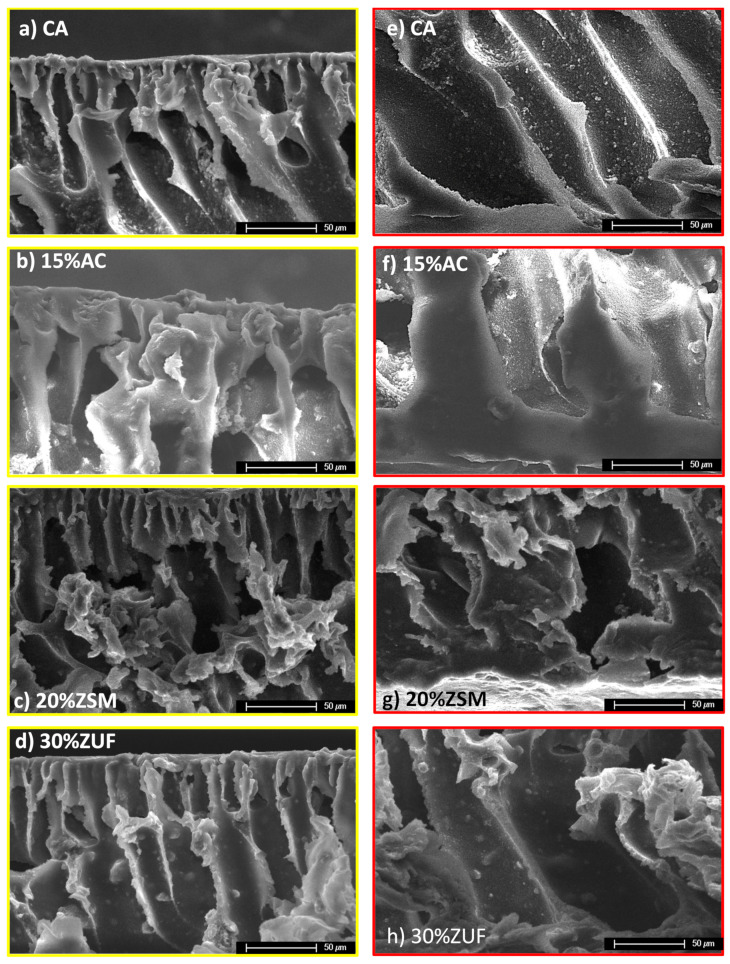
SEM images of pure CA membrane (**a**) top and (**e**) bottom; MMMA containing 15% of AC: (**b**) top and (**f**) bottom; MMMA containing 20% of ZSM-5: (**c**) top and (**g**) bottom; MMMA containing 30% of ZUF: (**d**) top and (**h**) bottom.

By zooming in the membranes (Figure 3) one can notice more in detail the effect of the filler addition on the pores. Yellow-framed pictures represent the top surface of the membranes (Figure 3a–d), while red-framed ones (Figure 3e–h) depict the bottom section in selected spots of the same membrane that are indicated in Figure 2.

The filler which affects more significantly the pore shape is, again, activated carbon, that increases the membrane tortuosity and closes, in some points, the pores at the bottom. The addition of ZSM-5 has a lower impact on the pore shape, although in certain zones of the membrane the bottom part of the pores is deformed and the pores show a dead end.

Finally, the crystalline structure of ZUF alters only slightly the membrane pores, making them narrower, but still well aligned both on the upper and lower surface. This is related to the small particle size, below 10 μm, of this filler which lies preferentially at the membrane bottom due to its higher density.

In conclusion, the effect of filler addition on the membrane morphology is affected by the particle size, density and chemical nature of the filler. The filler which a stronger impact on the membrane structure is activated carbon, while ZUF is the one which has a lower influence on the polymeric structure, due to a combination of the above factors. This behavior is probably due to the fact that the organic part of the membrane has a better affinity with the carbon particles as opposed to inorganic zeolites, and forms more interpenetrated composites. Moreover, the large size of the AC particles (>50 microns) and its lower density enable a more ubiquitous dispersion of the filler with respect to zeolites.

The SEM images allow also to detect the presence of the filler inside the membranes. In the case of zeolite-filled membranes, due to their chemical nature, the presence of particles can be assessed by the EDS analysis reported in Section S1 and Figure S3 of the Supplementary Materials. The qualitative EDS results performed on ZUF-based MMMAs confirm that the particles identified by SEM inspection have a chemical composition compatible with ZUF. In the case of AC-based MMMAs the particles are not clearly distinguishable via SEM due to their intimate combination with the polymer, and an EDS analysis would not be conclusive, but their presence is confirmed by the blackish color of the membrane, visible from Figure S4 in the Supplementary Materials.

#### 3.1.3. Density and Water Uptake of MMMAs

**Figure 4:**
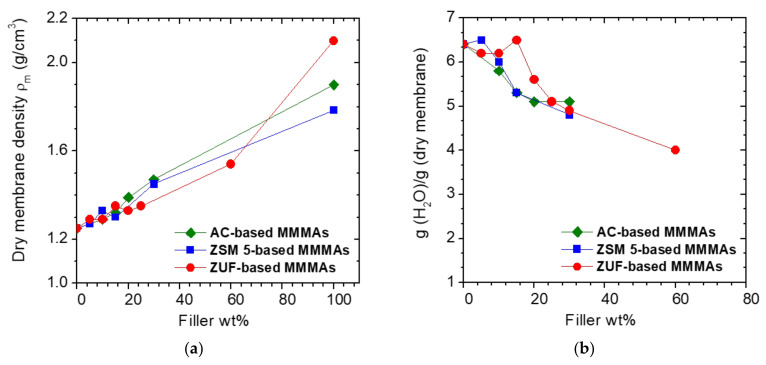
(**a**) Dry membrane density *ρ_m_* and (**b**) Water uptake, in grams of water per gram of dry membrane, versus filler weight percentage for the different MMMAs.

The MMMAs dry densities are reported in Figure 4a. The experimental point at 0 wt% of filler corresponds to the plain CA membrane, while the one at 100 wt% represents the filler density value from the literature [53,54,55]. Since the fillers are denser than the polymer, the MMMAs density increases with the filler loading in a rather regular way.

The water uptake of the MMMAs, in grams of solvent per gram of dry membrane, is shown in Figure 4b. The water uptake is not affected by the presence of filler at loadings below 5% and then starts to decrease, from 6.5 to about 4 g of water per gram of membrane at the highest filler loading. The decrease in water uptake can be due to the fact that all the fillers considered, namely activated carbons and activated zeolites, are more hydrophobic than the polymer matrix, but also to the fact that they modify the membrane structure. In particular, the water uptake capacity can be lowered by the general density increase of the MMMAs, measured through the density tests, but also by reduction of the membrane porosity by means of obstruction by filler particles. Indeed, we found that the reduced water uptake is correlated to the increased membrane density, as reported in the SI in Figure S5.

While the ability of the membranes to absorb water is reduced by the presence of fillers, the surface hydrophilicity of CA remains almost unaltered as indicated by the results of the contact angle analysis reported in Table S1. The values lie between 61 and 66° for all membranes. This indicates that, at least on the surface, the hydrophilicity of the pure cellulose acetate membrane is preserved and the presence of the adsorbents does not affect the wettability of the active membrane layer. Moreover, since the advancing and receding contact angles are practically the same, hysteresis is not observed. Since dynamic contact angle hysteresis is a measure of the surface roughness, its absence indicates that the surface of the MMMAs can be considered smooth without remarkable irregularities.

#### 3.1.4. Hydraulic Permeability of MMMAs

**Figure 5:**
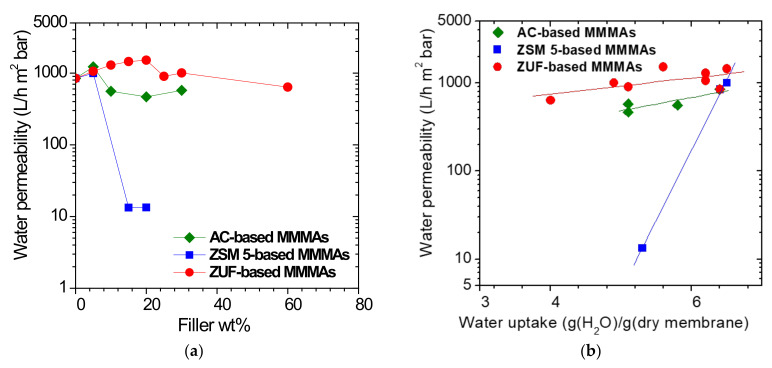
(**a**) Hydraulic permeability of MMMAs versus filler weight; (**b**) correlation between water permeability and water uptake in the different MMMAs.

The hydraulic permeability of the plain polymeric membranes and MMMAs decreases with time as a result of the gradual compression of the membrane pores due to the applied transmembrane pressure. An example of the time-evolution of the permeability is shown in Figure S6a in the Supplementary Materials, reporting data of the ZUF-based MMMA at various filler contents. It can also be noticed that the decrease of permeability with time due to the matrix compression, is less pronounced at higher ZUF loadings, due to the reinforcing effect of ZUF particles in the matrix. In Figure S6b we report a SEM picture of a CA membrane after the water permeability tests: it can be seen that, compatibly with the permeability results, the membranes undergo a certain compression which reduces their ability to permeate water with time. The data on the various membranes indicate that the permeability reaches a stable value after about 210 min of test. For this reason, the water permeability data measured are recorded after equilibration and reported in Figure 5a.

The permeability of the plain cellulose acetate membrane after 210 min is equal to 844 L/(h m^2^ bar). The permeability increases when adding a filler loading of 5wt%, for all the sorbent types considered, while for higher loadings the behavior diverges for different particle types. The permeability of MMMAs based on ZUF slightly increases continuously with filler content, reaching a maximum of 1513 L/(h m^2^ bar), and then decreases to 637 L/(h m^2^ bar) at very high filler loading (60wt%). On the other hand, the permeability of AC-based MMMAs increases to 1227 L/(h m^2^ bar) when the filler content is low (5%) and then decreases, becoming stable at values around 500 L/(h m^2^ bar) at 30% filler loadings. In the case of MMMAs based on ZUF the permeability increases to 993 when a 5% amount is added to the membrane, while it drops by almost 2 orders of magnitude for higher filler contents

The permeability data are in good linear correlation with the ones of water uptake (Figure 5b), indicating that the water flux in the membrane is correlated to the maximum amount of water that the membrane can uphold at equilibrium, as it is reasonable.

### 3.2. Batch Adsorption Tests

#### 3.2.1. Adsorbents

**Figure 6:**
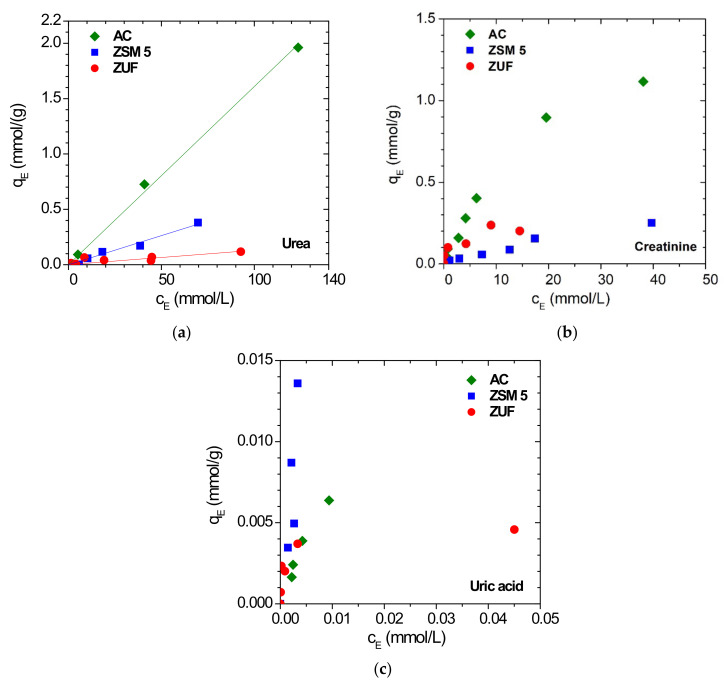
Static adsorption isotherms of (**a**) urea (**b**) creatinine (**c**) uric acid in the various fillers considered.

Since the goal of this project is the removal of uremic toxins from water, the static binding capacity of the adsorbents is explored in a range of toxins concentration going from c_N_ to c_MAX_ and the relevant isotherms are reported in Figure 6a–c for urea, creatinine and uric acid, respectively. The sorption isotherms are reported in terms of relative toxin uptake versus its concentration in solution at equilibrium, as the majority of the data reported in the literature for sorbent materials is displayed in this way. The urea adsorption isotherms show a linear behavior in all the three sorbents considered, with values which are higher for activated carbon and lower for zeolite ZSM-5 and ZUF, respectively. The adsorbents are endowed with different affinity for each toxin, indeed activated carbon is the most suitable adsorbent for urea and creatinine, while ZSM-5 has the highest adsorption capacity for uric acid. If we look at the comparison with previous data from the literature on activated carbon and ZSM-5, in Figure S7a,c for urea and Figure S7b,d for creatinine, respectively, we notice similar qualitative trends. However, the activated carbon inspected in this work has a higher average adsorption capacity with respect to the ones investigated in other works, maybe due to its higher surface area (1857 m^2^/g). In particular Cheng et al. used an AC sample with a total surface area of 978.5 m^2^/g while the sample of Kameda et al. had a surface area of 1433 m^2^/g [56]. The value given by Gelder et al. on the other hand, is a typical value measured in AC samples at a concentration of urea of 20 mmol/L, a value that is more than three times lower than the highest concentration possible in hyperuricemia conditions (c_MAX_).

#### 3.2.2. MMMAs

The MMMAs were tested for their batch adsorption properties according to the procedure described in Section 2.4.2. The results are reported in terms of toxin percentage removal, defined as the ratio between the mass of toxin adsorbed by the membrane at equilibrium to the mass of toxin present in the initial solution. This parameter represents an easier way to define the membrane ability to remove toxins in a real process.

The volume of MMMA used (8.83 × 10^−2^ mL) was the same for all experiments, while the value shown for the plain fillers (100 wt%) was measured on a different volume and were reported only for a qualitative comparison.

**Figure 7:**
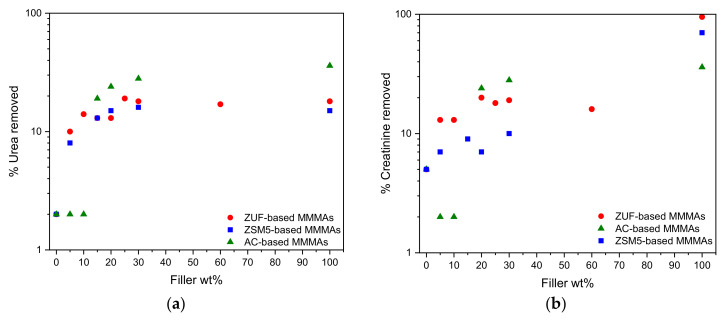
Percentage removal of (**a**) urea and (**b**) creatinine by the different MMMAs as determined in static adsorption tests.

In Figure 7a,b we reported the removal capacity of the MMMAs tested for urea and creatinine, while the complete data are reported in Table S2.

The removal of uric acid reached 97% in the plain cellulose acetate membrane, therefore the addition of fillers does not enhance the binding capacity for this toxin. This could be ascribed to the ability of uric acid to establish hydrogen bonds with the polymeric matrix. Its hydrogen bond donor count is the highest among the 3 toxins [42,43,44] and this promotes favorable interactions with the polymer, that exhibits a high hydrogen bound acceptor count [45].

The situation is completely different for creatinine and urea, where the removal of the plain CA membrane is very low compared to that of all the different MMMAs. In particular, adding the adsorbent to the membrane improves the urea removal capacity from 2% to factors as high as 19% when adding ZUF, 16% when adding ZSM-5, 28% when adding AC. Whereas for creatinine, the best combination is the one formed by CA with ZUF, followed by AC and ZSM-5 with factors that increase from 5%, for the plain membrane, to 20%, 10% and 21% when adding ZUF, ZSM-5 and AC, respectively. However, in the case of creatinine the removal enhancement is lower than expected, at least when adding zeolites to the polymer, as they have a high removal capacity for this toxin. A possible explanation is that the polymer may partially block the filler nanopores.

Nevertheless, MMMAs enhance the removal of urea and creatinine of the CA membrane as it can be observed in Figure 7a,b as a function of filler loading. The performance could be further improved by increasing the filler loading in the polymer matrix and/or by optimizing the preparation protocol and the membrane porosity.

### 3.3. Dynamic Adsorption Experiments

Preliminary dynamic binding experiments were performed with single toxins as to measure the dynamic binding capacity of the membranes and to compare the results with the ones obtained in batch tests. The dispersion curves necessary to calculate the dynamic adsorption capacities were obtained with NaCl, as it was found to be one of the few solutes not interacting with the membrane samples. Experiments were performed with a layered stack of 5 membranes corresponding to 0.7 mL of column volume and the results are reported in Figure S8. However, the membrane column volume was too small for the amount of toxins loaded and a bigger column volume was needed for the experiments with the toxin mixture. Therefore, 11 discs of MMMA_ZUF-60 and 11 of MMMA_AC-20, for a total of 2.7 mL of membrane column volume, were packed in the membrane holder. The adsorption tests were performed by feeding 40 mL of a mixture of the three toxins at their c_MAX_ at three different flow rates (0.5, 5 and 10 mL/min).

**Figure 8:**
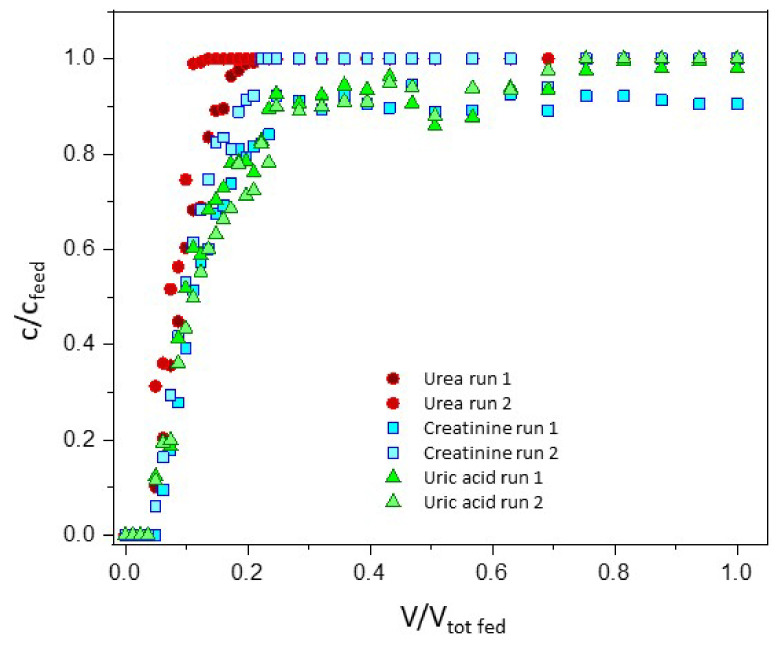
Breakthrough curves of urea, creatinine and uric acid for the MMMAs cartridge in contact with a mixture of toxins at 0.5 mL/min.

**Figure 9:**
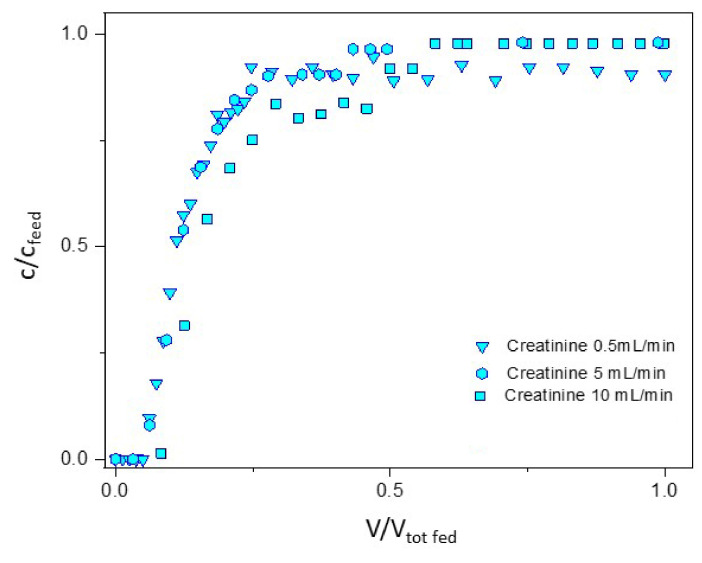
Breakthrough curves of creatinine for the MMMAs cartridge in contact with a mixture of toxins at the three different feed flow rates.

Figure 8 represents the breakthrough curves for the 3 toxins at 0.5 mL/min in duplicate experiments, while Figure 9 shows the breakthrough curves for creatinine at the three different feed flow rates.

The data were obtained by HPLC analysis of the collected fractions and, despite some scattering due to the manual integration of peaks, they are meaningful and reproducible. Urea is the limiting toxin for the separation since its breakthrough appears earlier than that of other toxins. From Figure 9 it is possible to see that the slope of the breakthrough curves is not affected by the increase of the flow rate from 0.5 to 10 mL/min, and thus that the diffusion limitations of the MMMAs are minimal.

Finally, we estimated the size of a cartridge able to remove uremic toxins from spent dialysate in a typical hemodialysis session of 3.5 h, with a dialysate flow rate of 450 mL/min. For the concentration of urea, creatinine and uric acid we used a mean value, and we fixed the percentage removal of toxins to 90%.

Based on the maximum binding capacity of each toxin evaluated from multicomponent tests in the multi-MMMA cartridge, it was calculated that a volume of 12 L of system would be required to obtain the specified removal of toxins accumulated in dialysate water during a typical HD session. Such value is largely determined by the removal of urea, that is the most abundant toxin and the most difficult to eliminate. Lower volume values could be obtained by optimizing the process configuration, and the sorbent materials.

## 4. Conclusions

In this work, MMMAs obtained by dispersing ZUF, ZSM-5 and AC in cellulose acetate were successfully fabricated via phase inversion and tested to evaluate their uremic toxin removal capacity. The SEM images highlight that the membrane porosity is disrupted when adding AC particles, while ZUF and ZSM-5 leave the pore morphology unaffected. The water permeability decreases with time ad reaches a stable value after approximately 210 min. Its value ranges between 600 and 1500 L/(h m^2^ bar) for the various MMMAs considered. The removal capacity of the membrane towards urea and creatinine in water is strongly enhanced by the presence of filler. Finally, the MMMAs were tested in dynamic experiments with a mixture of toxins in water, using a cartridge containing a combination of 22 membrane layers. The results indicate that the process is feasible and could be optimized by varying the configuration and the urea removal capacity of the sorbents.

The obtained results indicate the potentiality of porous materials and MMMAs in toxin removal for hemodialysis and justify further investigations. Future work will be devoted to find new materials able to achieve the desired separation and to increase the urea removal with functionalization of the polymer or the filler.

## Figures and Tables

**Table 1:** Properties of the most abundant uremic toxins in blood.

Toxin	MW (g/mol)	c_N_ (mg/mL)	c_MAX_ (mg/mL)	λ_UV-VIS_ (nm)
Urea	60.06	0.4	4.6	200
Creatinine	113.12	0.012	0.24	235
Uric acid	168.11	0.067	0.15	290

## Data Availability

The data presented in this study are available on request from the corresponding author.

## Supplementary Materials

The following are available online at https://www.mdpi.com/article/10.3390/membranes12020203/s1. Figure S1. Schematic of the (a) hydraulic permeability apparatus; (b) dynamic adsorption rig. Figure S2. Granulometry of (a) activated carbon; (b) Zeolite ZSM-5; (c) Zeolite Clinoptilolite ZUF used in this work. Section S1. EDS analysis of ZUF-based MMM. Figure S3. (a) EDS analysis of pure ZUF. (b) SEM image of the ZUF_MMMA membrane used for EDS inspection. (c) EDS analysis of the red circle of Figure S2b. (d) EDS analysis of the blue circle of Figure S2b. Figure S4. Pictures of the various MMMAs produced. Table S1. Advancing and receding contact angles of the various MMMAs. Figure S5. Equilibrium water uptake of the MMMAs versus dry membrane density. Figure S6. (a) Hydraulic permeability of MMMAs based on ZUF, versus time. (b) SEM image of a plain CA membrane after the permeability test. Figure S7. (a) Comparison between data obtained in this work on pure adsorbents and literature data. Table S2. Toxin removal in batch experiments with the different MMMAs. Figure S8. Breakthrough curves of (a) urea, (b) creatinine and (c) uric acid for a stack of 5 membranes (0.7 mL volume) obtained by feeding 40 mL at 00.5 mL/min of the pure toxin solution at c_MAX_.
