# Supplementary material for: Mixed Matrix Membranes Adsorbers (MMMAs) for the Removal of Uremic Toxins from Dialysate
Source: Membranes (Basel). 2022 Feb 9;12(2):203. doi: 10.3390/membranes12020203 (PMC8878186; doi:10.3390/membranes12020203)
Supplement: Supplementary file 1 [file membranes-12-00203-s001.zip › membranes-1557566-supplementary.pdf]

membranes

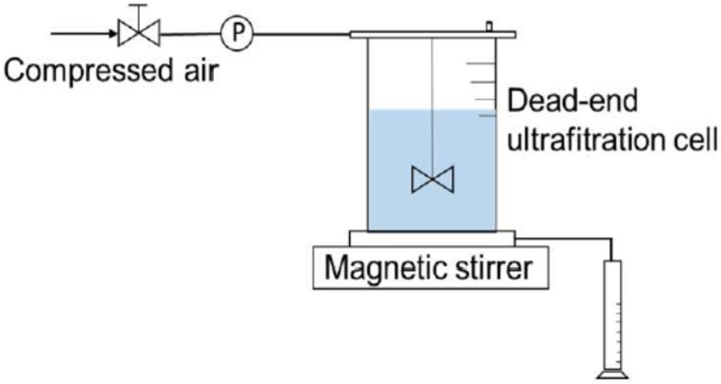

(a)

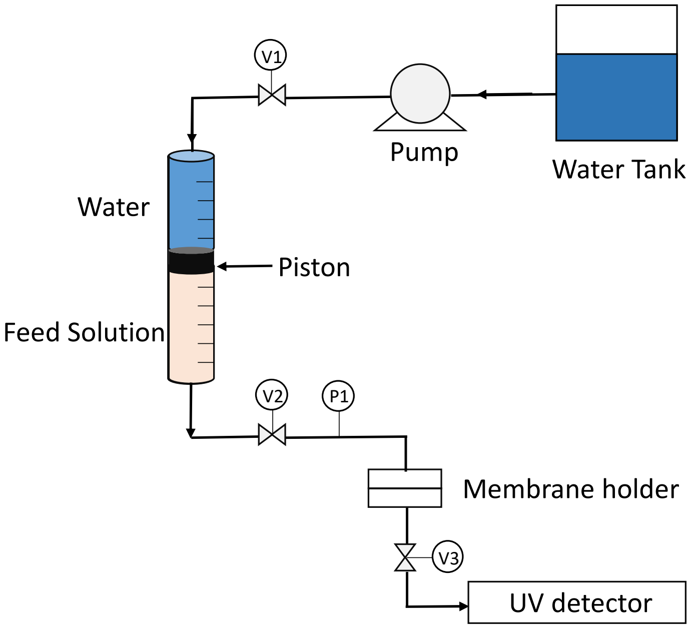

(b)

Figure S1. Schematic of the (**a**) hydraulic permeability apparatus; (**b**) dynamic adsorption rig.

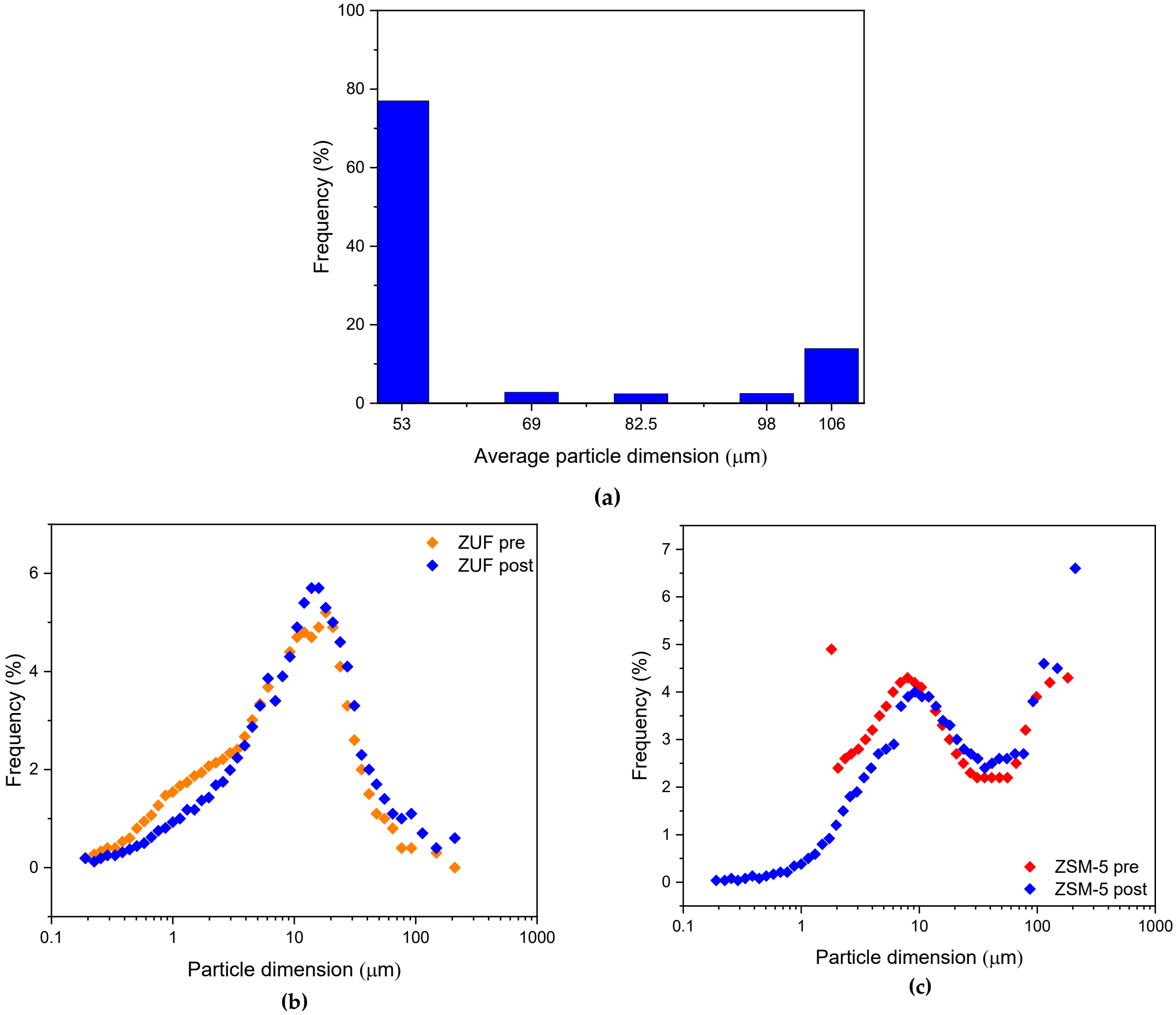

Figure S2. Granulometry of (**a**) activated carbon; (**b**) Zeolite ZSM-5; (**c**) Zeolite Clinoptilolite ZUF used in this work. "Pre" indicates the material before the activation treatment, "Post" the same material after the activation treatment.

S1. EDS Analysis of ZUF-based MMM

MMMAs based on ZUF were taken as example to assess the presence of the zeolite particles inside the matrix: due to similar chemical structure and granulometry with respect to ZSM-5, the analysis was considered representative of both zeolites. On the other hand, such analysis does not allow to reveal the presence of AC, as the peak related to carbon would be comprehensive of the signal of the cellulose acetate backbone and therefore not distinguishable.

The EDS carried out on pure ZUF (Figure S3a) reports the presence of silicon (Si), aluminum (Al), oxygen (O), potassium (K) and calcium (Ca), compatibly with its chemical structure. The spots on which the EDS was performed in a mixed matrix membrane containing 20wt% of ZUF are reported in Figure S3b. The results of the analysis on the circled spots (Figure S3c and d) confirm the presence of the zeolites in the MMMAs: in particular Si, O, K and Ca univocally identify the presence of ZUF. The Al signal, on the other hand, is due both to the aluminosilicate lattice and to the metallization.

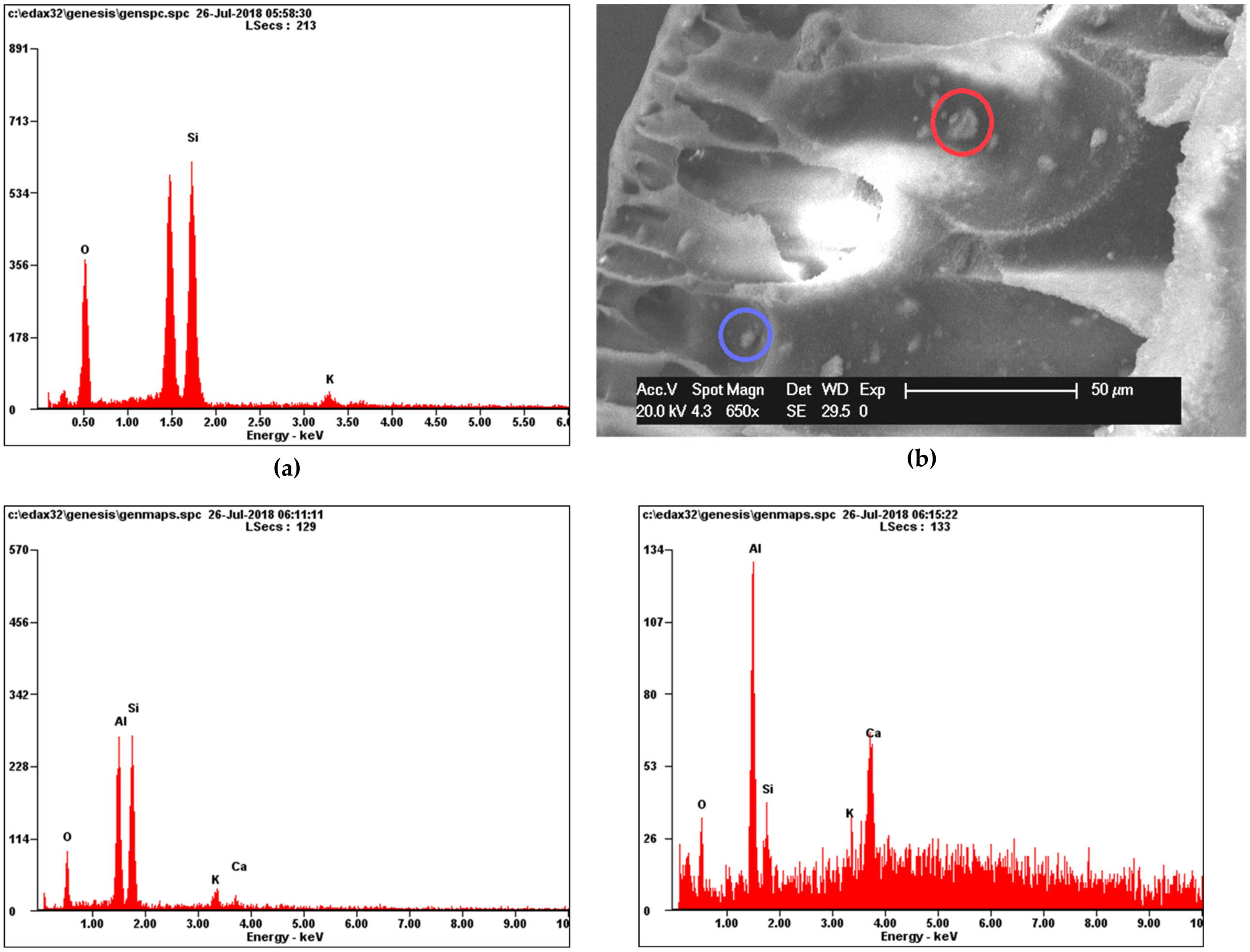

Figure S3. (**a**) EDS analysis of pure ZUF. (**b**) SEM image of the ZUF_MMMA membrane used for EDS inspection. (**c**) EDS analysis of the red circle of Figure S2b. (**d**) EDS analysis of the blue circle of Figure S2b.

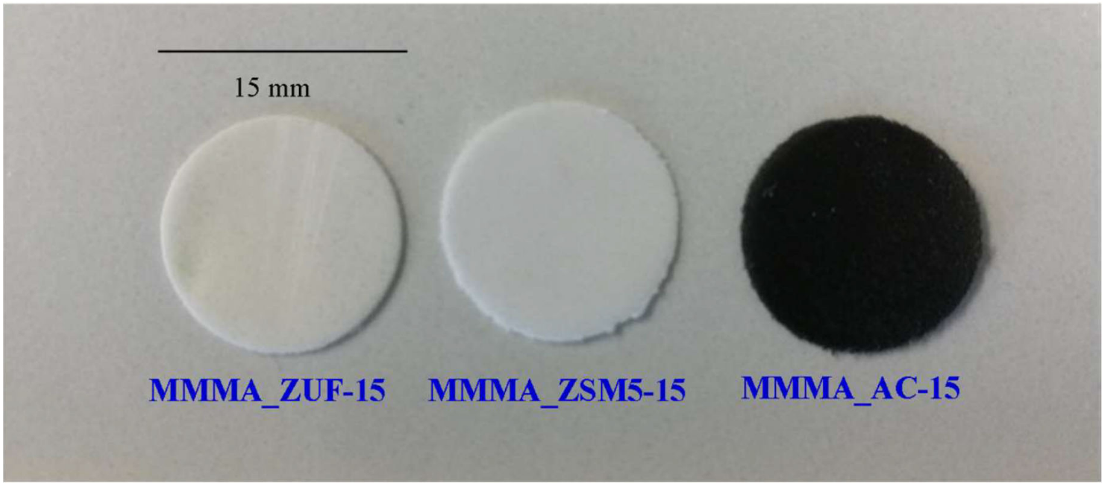

Figure S4. Pictures of the various MMMAs produced.

Table S1. Advancing and receding contact angles of the various MMMAs.

	Filler wt%	Advancing Contact Angle (°)	Receding Contact Angle (°)
MMMA-0	0	63	64
MMMA-AC	5	63	63
	10	65	66
	15	64	64
	20	63	64
	30	63	64
MMA-ZSM 5	5	62	63
	10	65	66
	15	65	66
	20	64	65
	30	64	65
MMA-ZUF	5	62	64
	10	66	66
	15	64	64
	25	61	62
	30	63	63
	60	64	65

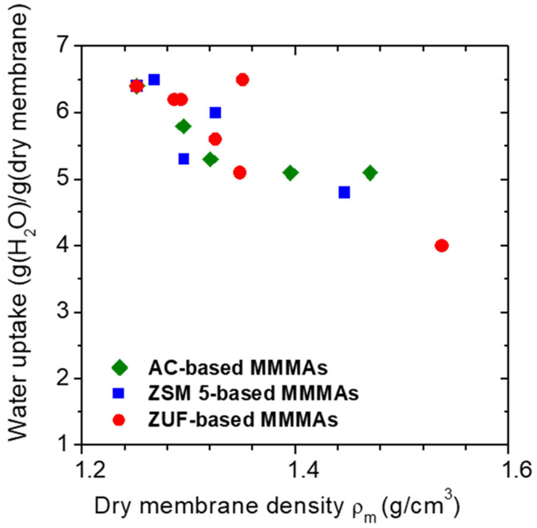

Figure S5. Equilibrium water uptake of the MMMAs versus dry membrane density.

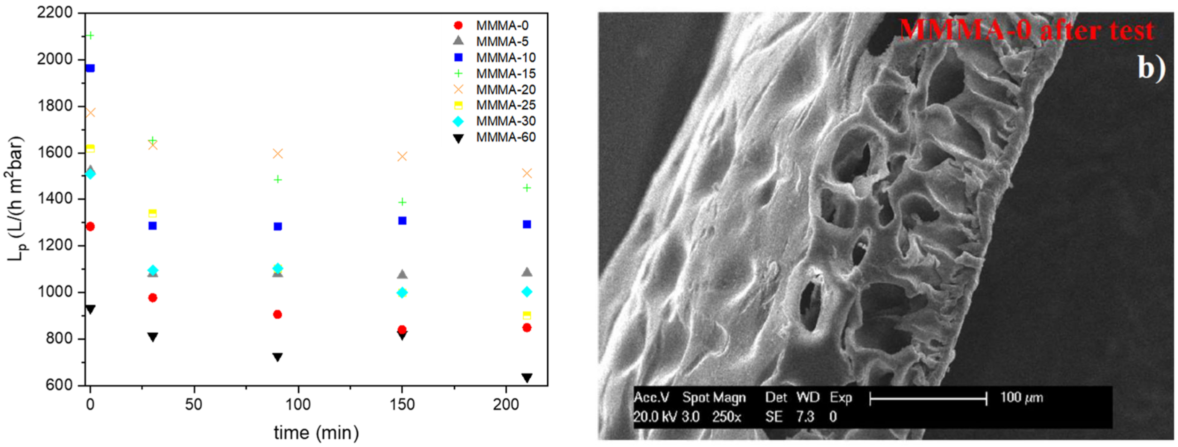

(**a**) (**b**)

Figure S6. (**a**) Hydraulic permeability of MMMAs based on ZUF, versus time. (**b**) SEM image of a plain CA membrane after the permability test.

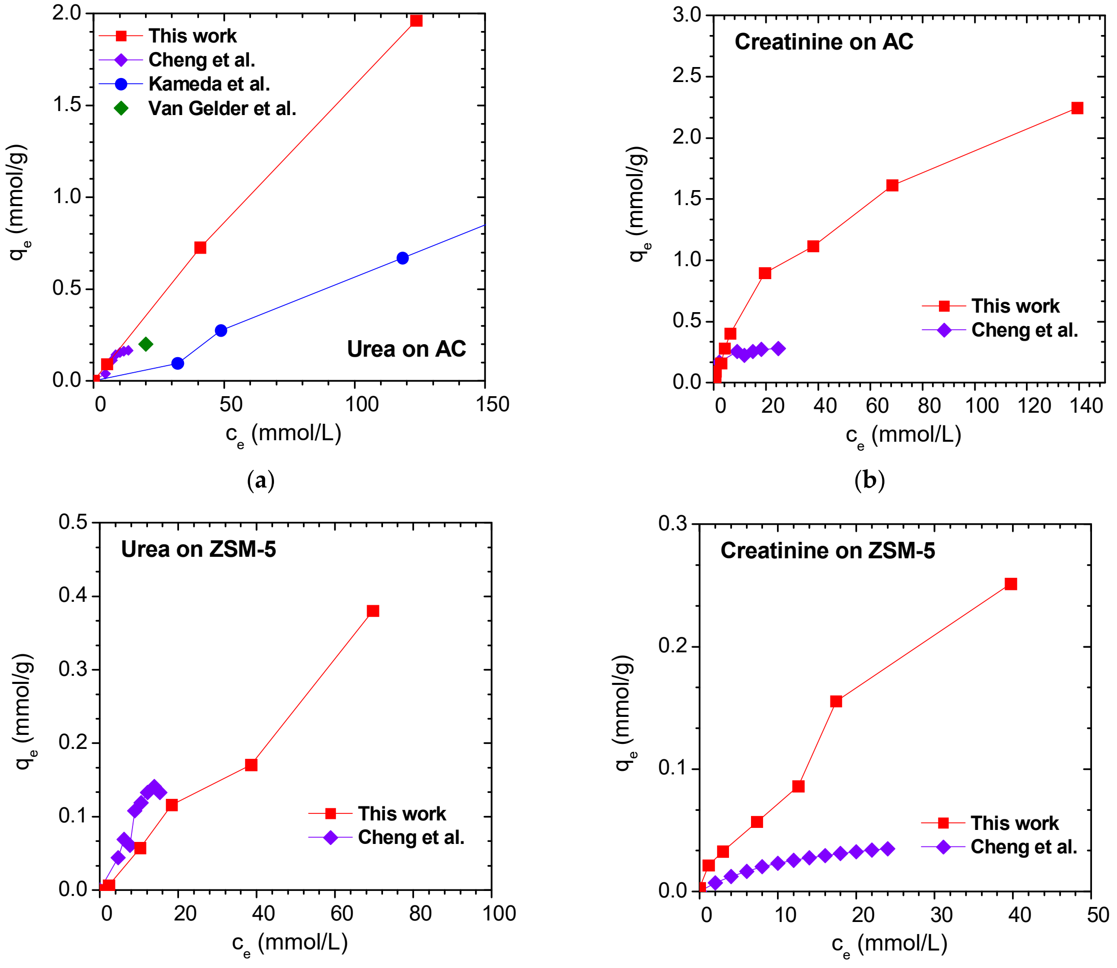

(**a**) (**b**)

(**c**) (**d**)

Figure S7. (**a**) Comparison between data obtained in this work on pure adsorbents and literature data from Cheng et al.(37°C) [27], Kameda et al. (30°C) [2], Van Gelder et al. [9]. **(a)** Urea adsorption in activated carbon, (**b**) Creatinine adsorption in activated carbon, (**c**) Urea adsorption in ZSM-5, (**d**) Creatinine adsorption in ZSM-5.

Table S2. Toxin removal in batch experiments with the different MMMAs.

Membrane	Urea Removed (%)	Creatinine Removed (%)	Uric Acid Removed (%)
MMMA-0 (plain CA)	2 ± 0.4	5 ± 0.4	97 ± 0.001
MMMA_ZUF-5	10 ± 0.5	13 ± 0.8	98 ± 0.001
MMMA_ZUF-10	14 ± 0.5	13 ± 1.2	97 ± 0.007
MMMA_ZUF-20	13 ± 3.3	20 ± 4.2	98 ± 0.431
MMMA_ZUF-25	19 ± 4.7	18 ± 8.15	96 ± 0.5
MMMA_ZUF-30	18 ± 9.7	19 ± 6	95 ± 1.46
ZUF (pure filler)	18	95	38
MMMA_ZSM5-5	8 ± 0.4	7 ± 0.33	90 ± 2.09
MMMA_ZSM5-15	13 ± 6	9 ± 0.54	95 ± 0.11
MMMA_ZSM5-20	15 ± 8	7 ± 1.98	98 ± 0.25
MMMA_ZSM5-30	16 ± 7.25	10 ± 5	97 ± 1.91
ZSM-5 (pure filler)	15	70	8
MMMA_AC-5	2 ± 1.62	14 ± 3.3	97 ± 0.5
MMMA_AC-10	2 ± 1.20	11 ± 1.33	92 ± 1.28
MMMA_AC-20	24 ± 1.2	10 ± 0.9	81 ± 1.2
MMMA_A-C30	28 ± 7	21 ± 2	89 ± 7.68
AC (pure filler)	36	37	98

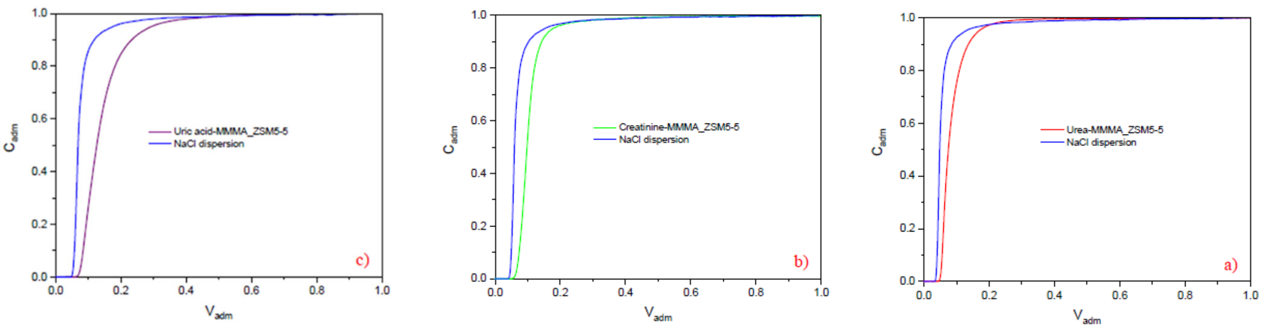

(a) **(b)** **(c)**

Figure S8. Breakthrough curves of (**a**) urea, (**b**) creatinine and (**c**) uric acid for a stack of 5 membranes (0.7 mL volume) obtained by feeding 40 mL at 0.5 mL/min of the pure toxin solution at cMAX. The corresponding NaCl system dispersion curves obtained 0.5 mL/min are also reported.
